# Platelet-Rich Fibrin Membrane for Pterygium Surgery: Literature Review and Feasibility Assessment

**DOI:** 10.7759/cureus.17884

**Published:** 2021-09-11

**Authors:** Carolina Camacho, Eduardo Rojas

**Affiliations:** 1 Ophthalmology Department, Universidad San Francisco de Quito, Quito, ECU; 2 “Incubadora de Investigación en Medicina" (InMed), NeurALL Nest, Quito, ECU; 3 Research Department, Medical School, Faculty of Health and Life Sciences, Universidad Internacional del Ecuador, Quito, ECU

**Keywords:** repair, ocular, surgery, platelet-rich membrane, pterygium

## Abstract

Pterygium is a common ocular disease caused by abnormal cellular proliferation leading to abnormal fibrovascular growth of the conjunctiva. The main treatment is surgical removal; however, despite the improvement of surgical techniques and development of adjuvant therapies, postoperative recurrence, which can be as high as 89%, remains a challenge. Currently, pterygium excision with conjunctival autograft remains the preferred surgical technique, although there is no gold standard technique to prevent pterygium recurrence. We have conducted a thorough and comprehensive review of the scientific literature regarding the use of PRF membranes in pterygium surgery. We aim to assess the safety, effectiveness, and applicability of platelet-rich fibrin membrane for primary pterygium surgery and assess its possible benefits in resource-limited settings.

## Introduction and background

Pterygium is an inflammatory, degenerative, and benign fibrovascular hyperplasia of the conjunctiva that extends across the limbus onto the cornea [1,2]. Nasal location is more common than temporal but, occasionally, can occur in both directions [1]. Even though its pathogenesis is not well understood, it is mainly associated with environmental factors, being ultraviolet radiation the major risk factor, which causes oxidative stress and hinders the normal cell proliferation cycle [1-3]. Genetic predisposition and viral infections (human papillomavirus and herpes simplex virus) have also been reported as causative factors [1,4]. Surgical removal is the only effective treatment; however, the risk of recurrence has been reported to be as high as 89%, depending on the technique used [1]. Recurrence is multifactorial depending on genetic, environmental, and surgical factors (surgical technique); hence, its incidence cannot be easily predicted [1,4].

Pterygium surgical treatment has evolved throughout the years, nowadays the “bare sclera technique”, which was the first adopted technique for pterygium removal, has been discontinued given its high rate of recurrence; thus, adjunct therapies have emerged to mitigate reappearance rates [3]. According to a recent meta-analysis, the best adjuvant treatment to reduce recurrence after pterygium excision is the association of a conjunctival autograft (CA) with ciclosporin 0.05% eye drops [3]. The rate of recurrence varies according to the technique used, but none have reported a lack of reappearance. As a result, there is a need to develop new therapeutic strategies that would decrease the proportion of repeat surgeries and therefore ameliorate the expense of such procedures to both patients and the healthcare system. 

One possible solution might lay within the patient itself, by using blood-derived products, rich in growth factors, to repair the surgical site after pterygium removal. Blood-derived products have been used in ophthalmology since 1946 [5]. Platelet-rich fibrin (PRF), described by Choukroun et al. in 2001, is a second-generation platelet concentrate obtained from centrifugation of an autologous blood sample and from which we obtain a PRF membrane enriched with platelets and their biologically active agents that favors hemostasis, epithelial regeneration and wound healing at the injury site [1,2,5,6].

This study aims to analyze the safety, effectiveness, and applicability of PRF membrane for primary pterygium surgery through literature.

## Review

Why not use what is already there? The benefit of blood-derived products

Blood-derived products have been used in ophthalmology since 1946 [5]. Platelet preparations are an autologous source of growth factors, adhesion molecules, cytokines, and hemostatic factors; hence, platelets enhance cell adhesion and proliferation, aiding in the regeneration of the ocular surface epithelium [2,5,7]. They also have the anti-inflammatory, antifibrotic, and antimicrobial potential [8]. PRF, described by Choukroun et al. in 2001, is a second-generation platelet concentrate obtained from centrifugation of an autologous blood sample and from which we obtain a PRF membrane, which consists of 3-D polymerized autologous fibrin matrix enriched with platelets and their biologically active agents that favors hemostasis, epithelial regeneration and wound healing at the injury site [1,2,5,6].

PRF application has been widely described in dentistry, orthopedics, plastic surgery, and otorhinolaryngology. However, its use in ophthalmology is relatively new, being mostly used in the management of corneal and ocular surface pathologies [2,8]. For example, a Randomized Controlled Trial that assessed the use of PRF membrane for the repair of palatal donor-free gingival grafts and compared it with commercial collagen dressings (CollaCote®), reported no significant difference between the use of both procedures [9]. Furthermore, the PRF membrane was easier to use and resulted in significantly fewer costs as compared with the commercial option [9]. Moreover, apart from its use in the mucosal epithelium, PRF has also shown promise in nervous tissue and animal models of peripheral nerve injury [10]. 

However, the success of this technique entirely depends on the time gap between blood collection and its transfer to centrifuge because PRF polymerizes naturally and slowly during centrifugation. Therefore, centrifugation protocols must be standardized since this process has a significant impact on the biological properties of the fibrin matrix [6,11].

From blood to membrane: building the fibrin scaffolding

PRF is a second-generation platelet concentrate described by Choukroun et al. [2]. Platelets are natural reservoirs of growth factors such as epidermal growth factor, fibroblast growth factor, transforming growth factors, platelet-derived growth factors, vascular endothelial growth factors, and insulin-like growth factor; cell adhesion molecules like fibrin, fibronectin and vitronectin, and thrombospondin; and cytokines [2,5,12]. Cell adhesion molecules enhance growth factor activities [12]. During platelet activation, these factors are released at the site of injury facilitating wound healing [5,12].

**Figure 1:**
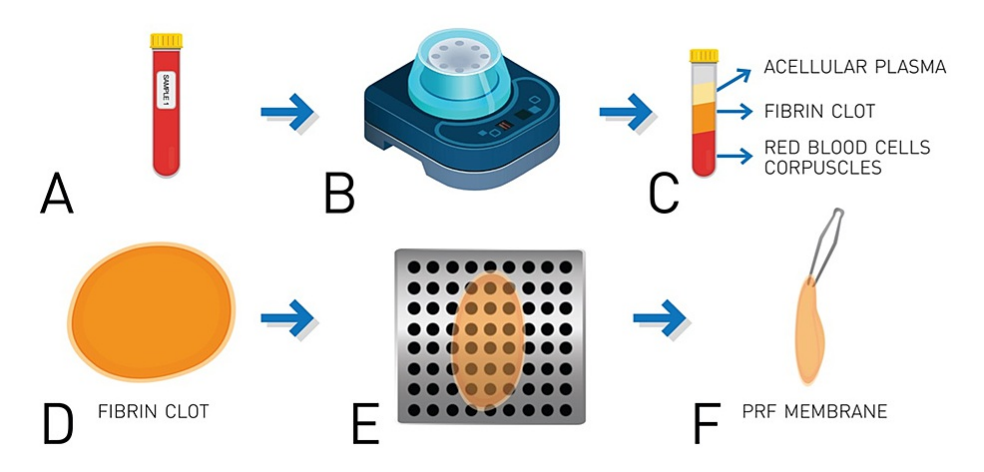
Process of PRF membrane construction (A) Venous blood sample is collected from the patient in a sterile, coagulant-free recollection tube. (B) The tube is centrifugated at 3,000 rpm for 10 minutes. (C) This results in the concentration of fibrinogen in the superior two-thirds of the tube and its polymerization in a fibrin clot (D). (E) The clot is carefully drained to finally obtain the platelet-rich fibrin (PRF) membrane (F).

PRF membrane is a three-dimensional, biocompatible, biodegradable biopolymer, which is obtained through a simple protocol and does not require any additives [6,7]. Its preparation requires the collection of a venous blood sample, without anticoagulant, in a sterile tube which is immediately centrifugated at 3000 rpm for 10 minutes [6,13]. Centrifugation concentrates fibrinogen in the middle and upper parts of the tube, the absence of anticoagulant allows platelet activation with the resulting transformation of fibrinogen into fibrin, which polymerizes to a three-dimensional fibrin mesh; platelets and leukocytes are trapped in this mesh [6,7]. A fibrin clot is formed in the middle of the tube, which is drained to obtain the PRF membrane that acts as a physiologic fibrin matrix scaffold, which interacts with the patient’s cellular matrix and sustainably releases epitheliotropic factors to support cell adhesion, proliferation, migration and differentiation of the ocular surface epithelium; hence, promoting healing [6-8,13]. A pictorial representation of this process can be found in Figure 1.

The PRF membrane can retain, for over a week, almost 30% of growth factors and its natural fibrin framework properties allow growth factors to keep their activity for a longer period [2,7,8,12]. As the fibrin matrix degrades, a progressive and controlled release of epitheliotropic factors occurs at the site of injury, which enhances cell proliferation and adhesion; hence, the effect on wound healing is stronger and durable [2,7,8,12]. PRF constitutes a scaffold not only for growth factors but also for migrating endothelial cells; this combination of mechanical and chemotactic support highlights its potential for ocular surface reconstruction [2].

Bridging the gap: the application of PRF in pterygium surgery

Treatment of pterygium is essentially surgical; however, high rates of postoperative recurrence remain a challenge. Recurrence is associated with pterygium type and size, age of the patient, environmental factors, and surgical technique, surgical trauma, postoperative inflammation, and fibroblast proliferation [2,4]. It has been reported that 50% of recurrences occur within the first four months, while 97% appear within the first 12 months [1].

The “bare sclera technique alone” is no longer recommended due to its high recurrence rates, being as high as 89% [1]. Therefore, new adjunctive therapies have emerged; of these, a CA is the most commonly used technique due to its low recurrence risk of 5 to 10% and adequate safety margin [1,14,15]. A recent meta-analysis by Fonseca et al. that compares the effectivity of 14 adjunct therapies for pterygium excision concluded that conjunctival autograft with ciclosporin 0.05% eye drops was most effective in preventing postoperative recurrence [3]. However, this meta-analysis did not include the use of PRF membrane and included only studies assessing either CA or bare sclera technique, with and without adjuvant treatments [3]. Disadvantages of the use of adjuvant therapies such as ciclosporin, mitomycin C, beta therapy, 5-fluorouracil, and anti-vascular endothelial growth factor include potentially adverse effects and high costs; nowadays beta radiation is barely used due to its potentially devastating complications [4,14,16].

PRF membrane has exhibited favorable clinical results in the treatment of ocular surface pathologies, mainly corneal ulcers [8]. Nevertheless, there are few reported studies on the use of PRF membrane in pterygium surgery. To the best of our knowledge, there are only two studies related to the use of PRF membrane for pterygium surgery. These compared the use of CAs and PRF membrane. Cakmak et al. evaluated 35 patients' surgical time, complications, conjunctival epithelization time, suture reaction, postoperative inflammation, and recurrence rates. In this study, PRF membrane was superior to CA by a shorter surgical time (about 10 minutes) and milder postoperative inflammation; no suture reaction was observed with PRF membrane; however, the difference was not statically significant, which might be explained by the small sample size [2]. On the other hand, Zhao et al. evaluated 62 patient’s surgical time, complications, and recurrence rates. Surgical time was shorter for PRF membrane, no significant differences were found between techniques in complications and recurrence rate [17]. These studies concluded that PRF membrane is a safe, effective, and promising method for pterygium surgery with low rates of recurrence and complications [2,17]. It is important to note that postoperative inflammation has been related to pterygium recurrence [2]. PRF membrane facilitates tissue regeneration after ocular surface surgery thus minimizing inflammation [8]; moreover, shorter surgical time reduces ocular surface manipulation reducing postoperative inflammation, hence reducing recurrence [2]. We believe that PRF membrane could be a potential treatment option that might result in a lower economic burden for low-income patients and underdeveloped countries due to its ease of use and potential reduction of surgical time in the operating theater.

Nonetheless, these are preliminary studies with important limitations such as sample size and statistical power. Further studies are required to completely assess the benefits of this technique in the long term and completely assess its economic benefit for both the patients and the healthcare system. Table 1 summarizes the advantages and disadvantages of pterygium repair using PRF versus other techniques.

**Table 1:** Comparison of pterygium surgical techniques

Technique	Advantage	Disadvantage
Conjunctival autograft [4]	Easy to perform, low recurrence rates	Greater postoperative, inflammation, greater suture reaction, possible graft loss, longer surgical time, possible pain and discomfort
PRF membrane [2,17]	Easy to perform, low recurrence rates, shorter surgical time – decreased costs Less postoperative inflammation, Less suture reaction	Possible graft loss, Possible pain and discomfort
PRF membrane + Conjunctival autograft [2,4,17]	Easy to perform, Low recurrence rates	Moderate postoperative, inflammation, Greater suture reaction, Possible graft loss, Longer surgical time, Possible pain and discomfort

Pterygium prevalence in our country, Ecuador, is not well documented; however, small cross-sectional studies have reported a prevalence ranging between 9% and 55.4% [18-20]. Considering that the basic monthly salary, determined by the Ecuadorian Ministry of Labor for 2021, is 400 USD and that pterygium surgery ranges from 600 to 2,500 USD per eye; the need for cheaper alternatives with similar results is something warranted [21]. Certainly, a good proportion of underdeveloped countries face the same reality, which is why health equality, especially regarding illnesses that require surgical treatment, is a major concern in these areas of the world. Since the use of PRF membrane for pterygium surgery has shown comparable results and it has been associated with shorter surgical time, hence less cost, it is a promising method for resource-limited countries.

Limitations

There is very limited evidence available in the use of PRF is not widespread. Therefore, evidence is of low quality and very limited in regard to sample size. Nonetheless, we believe that the use of PRF membrane shows promise, and more studies should be performed to elucidate its long-term outcomes as well as assess its potential in resource-limited settings, in terms of saving economic and hospital resources.

## Conclusions

PRF membrane is a promising method with low rates of recurrence and complications.

Previous studies have yielded positive preliminary results and are the foundation for future investigations. A clear advantage of this method is the use of autologous material for surgery, it is time-saving and easy to prepare; therefore, PRF membrane is a safe, economic, effective, and feasible technique for pterygium surgery that should be further evaluated and validated.
